# Absence of Evidence as The Evidence Of Absence: The Curious Case of Latent Infection Causing Ocular Tuberculosis

**DOI:** 10.3389/fopht.2022.874400

**Published:** 2022-05-02

**Authors:** Soumyava Basu

**Affiliations:** Prof Brien Holden Eye Research Centre, LV Prasad Eye Institute, LV Prasad Marg, Hyderabad, India

**Keywords:** ocular TB, latent infection, pulmonary TB, TB immunoreactivity, tuberculin skin test, interferon gamma release assay

## Abstract

Ocular tuberculosis (TB) is frequently considered as intraocular inflammation in the setting of latent TB, owing mainly to the absence of microbiological evidence of *Mycobacterium tuberculosis* in ocular fluid samples. Even though such lack of microbiological evidence, and of systemic signs of active TB disease, are suggestive of latent TB infection, molecular and rare histopathologic evidence of mycobacteria in the eye, and favourable response of ocular inflammation to anti-TB therapy point to the presence of active infection in ocular TB. Here, we discuss how intraocular inflammation in ocular TB is not merely an immunologic response to bacilli, but an active tuberculosis infection. We will discuss the reason for the frequent absence of microbiological evidence of TB in the eye in ocular TB and the diagnostic hierarchy to arrive at the diagnosis of this infectious uveitis entity.

## Introduction

Let us think of two of the greatest scientific theories in human history − the theory of evolution and the Big Bang theory. While there are several pointers to their existence, there is no direct proof for either of them. Yet, scientists across the world continue to believe in them and are engaged in cutting edge research based on these theories. It would be reasonable to state, that in science, the absence of evidence does not always imply the evidence of absence. Although it may appear counterintuitive, the above aphorism can be applied to the practice of evidence-based medicine (EBM) too. This assumption is based on the fact that EBM is not merely “cookbook” medicine based on a compendium of irrefutable evidence (1). Instead, EBM requires the integration of clinical expertise with the “best available external evidence”. The external evidence can be drawn from the basic sciences, and from studies on diagnostic accuracy and therapeutic efficacy. In this way, the absence of evidence such as randomized clinical trial does not necessarily amount to the evidence of absence in the practice of EBM.

One condition where the above reasoning can find application in ophthalmology is ocular tuberculosis (TB). It’s causative organism, *Mycobacterium tuberculosis* (*Mtb*) was first identified in the eye in 1883, only a year after Robert Koch first discovered the bacillus (2). Yet ophthalmologists continue to associate the clinical manifestations of ocular TB with latent *Mtb* infection in the patient (3–5). It is assumed that the absence of microbiological evidence of *Mtb* infection in the ocular fluids of these patients, is sufficient evidence for the absence of mycobacterial infection in these eyes. Put differently, the absence of evidence becomes the evidence of absence. Since these patients have immunological and/or radiological evidence of systemic TB infection, but no active pulmonary TB, it is also assumed that the manifestations of ocular TB are associated with latent TB infection. This has led to a unique situation; wherein ocular TB is possibly the only human infectious disease that is associated with a latent infection.

In this perspective, we will first understand the divergence between the concepts of ocular TB and latent TB, and how intraocular inflammation could be induced by *Mtb* despite its absence in ocular fluids. We will then analyze how ocular TB [and other extrapulmonary TB (EPTB)] are not always associated with active pulmonary TB (PTB). Finally, we will focus on the tests for TB immunoreactivity – tuberculin skin test (TST) and interferon gamma release assays (IGRA) – and discuss ways to interpret them meaningfully for the diagnosis of ocular TB. Some of the terms that have been used frequently in this review have been explained in Box 1.

## What is Ocular TB?

The past two decades have seen a significant evolution in our understanding of ocular TB. During this period, ocular TB has been defined by clinical criteria that include characteristic ocular signs, ancillary evidence of systemic TB infection (immunological or radiological tests) and the exclusion of non-TB entities, and *not necessarily* histopathological or microbiological evidence of TB (6, 7). In broad terms, this definition has been endorsed by the multinational Collaborative Ocular Tuberculosis Study (COTS) (8), as well as the Standardization of Uveitis Nomenclature (SUN) Working Group (9). The range of ocular signs identified as ocular TB is wider and more inclusive in *diagnostic criteria* (such as the COTS), while it is more restricted in the *classification criteria* recently published by the SUN Working Group. In addition, the COTS group has termed the disease as “ocular TB” to connect the anatomical structure involved (eye) with the pathogen (*Mtb*) that requires specific anti-microbial therapy.

Despite the availability of these nomenclature and diagnostic criteria, alternative definitions of ocular TB continue to appear in ophthalmic literature from time to time. The terms “TB-uveitis” or “TB-associated uveitis” have been used selectively for patients with uveitis and active systemic TB, or when the intraocular inflammation resolves with anti-TB therapy (ATT) alone (12, 13). ‘Active’ ocular TB has been used in the context of patients microbiological or polymerase chain reaction (PCR) evidence of *Mtb* in ocular fluids (13). The remaining patients, who comprise the majority, and neither have active systemic TB nor any evidence of *Mtb* in ocular fluids, have been labelled as uveitis associated with latent TB infection (3–5, 12, 13).

## Latent TB Infection and Its Incompatibility with Ocular TB

The World Health Organization (WHO) defines latent TB as a state of persistent immune response to stimulation by mycobacterial antigens without evidence of clinically active manifest TB (10). It makes no reference to the biological state of the organism within the host. If we consider microbiologically proven TB alone as “active manifest TB”, then most ocular TB patients who present only with immunological evidence of TB, and not microbiological evidence, will be labelled as latent TB. However, this assumption misses on several important facts that are not congruent with latent infection. Firstly, ocular TB frequently resolves (or does not recur) following treatment with ATT indicating the presence of actively proliferating mycobacteria (3–5). Secondly, granulomatous inflammation and molecular/microbiologic evidence of *Mtb* that have been reported in various clinical subtypes of ocular TB (14), are representative of the immune reaction against *Mtb* bacilli and cannot be explained by latent TB infection.

The final argument against latent TB infection in ocular TB is related to the current understanding of the bacterial state in latent infection. The standard narrative for TB has been that a third of the world’s population has latent infection with *Mtb*, and 5-10% of those infected develop disease due to ‘reactivation’ of the latent infection (10). The latent infection is defined by TB immunoreactivity – TST or IGRA (in the absence of clinical disease) – and it is therefore convenient to label anyone with positive TST/IGRA results and no ‘obvious’ TB disease as latent TB. This narrative has now been challenged through literature review of epidemiological data, from the pre- and post-antibiotic eras (11, 15). It has been shown that most TB disease occurs within 18-24 months of infection, and there is no special bacterial state (such as dormancy) during the asymptomatic phase of TB. In fact, in both latent (asymptomatic) and active TB, the organisms appear to be a mix of active and non-replicating bacteria, and the metabolic state during latency is like the replicative state (16). In summary, the presence of ATT-responsive intraocular inflammation, and the timelines of progression from *Mtb* infection to disease, effectively rule out any role of latent infection in the pathogenesis of ocular TB.

It needs to be emphasized here that the presence of TB immunoreactivity due to memory T-cells does not necessarily indicate presence of viable bacteria in the body. Memory T-cells are maintained by a slow, but steady process of self-renewal that is not antigen-dependent (17). While these cells may themselves be relatively short lived (30-160 days), the immunologic memory tends to last longer (half-life of 8-15 years) (18). The implication for ocular TB is that the mere presence of positive TST/IGRA tests does not guarantee the presence of viable *Mtb* in the patient. Additionally, the elimination of bacteria by the host immune response or by ATT will not influence the TST/IGRA outcomes in the patient.

## Possible Mechanisms of Ocular TB in the ‘Absence’ of Microbiological Evidence of *Mtb* Infection

There are several explanations to the absence of *Mtb* in the ocular fluids, and of active systemic TB, in most cases diagnosed as ocular TB. The best explanation for both the above observations can be obtained from the available histopathological studies of ocular TB specimens. Here are key points that emerged from a clinicopathological study of enucleated eyes from 42 patients that were histopathologically proven to be ocular TB (19).

1. Acid fast organisms were found in 37 of 42 specimens in ocular or ocular adnexal structures. The ocular specimens were typically paucibacterial with only 1-2 organisms noted in the entire specimen, mostly in the vicinity of giant cells or an area of necrosis.
2. Nine of 42 also had *Mtb* in other organs, four of which were *only in* extrapulmonary organs.
3. Seven (40%) of the 17 available tuberculin skin test (TST) reports were negative, while eight (57%) of 14 chest radiographs were normal.

These data clearly explain why microbiological or even molecular evidence of *Mtb* is rarely found in aqueous or vitreous samples of patients with ocular TB. If the organism is so sparse in the ocular tissues, it would be even more unlikely in the adjacent ocular fluids. Additionally, the blood retinal barriers (even if partially disrupted due to the inflammation) would restrict the passage of organisms from the underlying tissues into the ocular fluids.

An obvious question that emerges from this data is what drives the widespread inflammatory response in the eye, if such few *Mtb* are present in ocular tissues. Current evidence points at two possible mechanisms that are not directly related to actively replicating Mtb. The first one is *autoimmunity* against retinal antigens. Flow cytometric studies of vitreous T-cells of patients with ocular TB, have revealed a highly pro-inflammatory cytokine response against retinal autoantigens, apart from that against *Mtb* antigens (20). How *Mtb* infection triggers the autoimmune response in the eye is not clear, but it is likely that the autoimmunity augments the inflammatory response in the eye. The role of autoimmunity has also been highlighted in TB affecting other organs, including pulmonary TB (21). For example, in the lungs, the extensive pathology far outweighs the number of bacteria found in the tissues.

The second possible mechanism is the inflammation induced by *bacterial products.* There are several indications to the existence of this mechanism. In experiments performed nearly a century ago, injection of heat-killed *Mtb* into internal carotid arteries of rabbits could induce inflammation in nearly all ocular tissues (22). Furthermore, heat-killed *Mtb* has been used as component of Complete Freund’s Adjuvant, along with retinal autoantigens for inducing experimental autoimmune uveitis (23). More recently, *in vitro* and animal studies have demonstrated innate immune response in the retinal pigment epithelium to mycobacterial antigen and double-stranded RNA (24). Together, it appears that replicating Mtb, even if present in numbers too few to be detected in the ocular fluids, can be supported by a retinal autoimmune response, and by bacterial products, in inducing intraocular inflammation.

## Absence of Systemic TB in Patients with Ocular TB

Since microbiological evidence of ocular TB infection is rarely found, an evidence of systemic TB infection, is critical to the diagnosis of ocular TB. This could either be an active TB disease (PTB or EPTB), or as in most cases, immunoreactivity to TB antigens. As noted above, even in histopathologically-proven ocular TB, active systemic TB may *not* be present in all cases (19). This phenomenon is not unique to ocular TB alone. Even among other forms of EPTB, PTB has not been found in more than half the cases (25). Hence the question arises, how does ocular TB, or any other EPTB, occur in the absence of pulmonary disease. The answer probably lies in the extrapulmonary niches of *Mtb* infection that exist in nearly every organ of the body (26). These include both professional phagocytic cells and other intracellular niches, present in different organs. Among these, the bone marrow stem cells (mesenchymal and hemopoietic) are of particular interest, since they not only harbor the infection, but also disseminate it to other parts of the body. In the eye, the retinal pigment epithelial (RPE) cells may have a significant role, since they can phagocytose *Mtb,* to a similar extent as macrophages (27). Mtb-laden RPE cells survive longer in the presence of *Mtb* as compared to traditional phagocytes, thus becoming ideal reservoirs of the infection in the eye (27). To summarize, *Mtb* can exist and disseminate within the host, in the absence of pulmonary disease. Absence of PTB in a suspected case of ocular TB should not deter the diagnosis, rather it should stimulate a search for EPTB elsewhere in the body. The relative importance of each of the types of evidence of *Mtb* infection that are used in diagnosis of ocular TB is given in Box 2.

## How Do We Interpret the Available Evidence for the Diagnosis of Ocular TB?

The supportive evidences for the diagnosis of ocular TB (Box 2) follow a hierarchy, whereby the strongest possible evidence is also the rarest in clinical practice. In this hierarchy, microbiological evidence of *Mtb* in ocular tissue or fluid samples remains the strongest, yet the rarest association of mycobacterial infection with intraocular inflammation. Similarly, the amplification of mycobacterial DNA from ocular samples, or the presence of active PTB or EPTB in patients with relevant ocular signs, are diagnostic of ocular TB, unless there is clear evidence for an alternative diagnosis. However, in clinical practice, the immune response to *Mtb* antigens as tested by TST *(in vivo)* or IGRA *(in vitro)* remains the most commonly used evidence, for the diagnosis of ocular TB. These tests measure the memory T-cell response to *Mtb*, that follows the development of adaptive immunity against the organism. However, neither test is able to distinguish between infection and active disease, or between present and past infection. As noted above, the presence of TB immunoreactivity does not necessarily confirm continued TB infection in the body (15). Hence, the utility of these tests in the diagnosis of ocular TB is closely linked to the context in which they are performed. This includes the presence of appropriate clinical signs and exclusion of non-TB entities. In mathematical terms, the value of the context in which a diagnostic test is applied is measured by the Bayes’ theorem (31). With this theorem, the pre-test probability of the disease (prevalence in general population), and the sensitivity and specificity of that test, are used to calculate the post-test probability of having a disease after the test is performed.

What might be the impact of the Bayes’ theorem on the interpretation of TB immunoreactivity for the diagnosis of ocular TB? Firstly, it means that these tests cannot be used in isolation for the screening of ocular TB, especially in low endemic populations. For example, a study from the United States showed that the post-test probability of ocular TB is only 1% in a patient with uveitis and a positive TST (32). However, the post-test probability increased to 30.3%, when TST was used for screening foreign-born patients for ocular TB in the United States (33). In high endemic populations too, the clinical presentation will influence the pre-test probability and thereby the post-test probability of ocular TB. For example, in a patient with non-granulomatous anterior uveitis with inflammatory joint pain, or with bilateral exudative retinal detachment suggestive of Vogt-Koyanagi-Harada disease, a positive TST will be of little value even in a TB-endemic country. Conversely, a higher pre-test probability of TB disease will lower the cut-off value for a positive test. Thus the Centre for Disease Control recommends a TST cut-off value of 15 mm of induration for non-endemic countries and 10 mm for TB-endemic countries (34). The cut off is even lower at 5 mm if there is a history of recent TB contact, previous TB disease or immunosuppression (e.g. HIV, organ transplantation). It will be interesting to investigate if this analogy can be applied to highly predictive signs of ocular TB such as serpiginous-like choroiditis, to lower the cut-off value in a given population. Table 1 highlights some of the common myths and facts associated with TB immunoreactivity tests.

A more difficult situation arises when the TB immunoreactivity tests are negative despite the tell-tale signs of TB in the eye. This can be a challenge since these tests are often the only link connecting the ocular disease to TB infection. It has been demonstrated that even in culture confirmed PTB, 10-40% of HIV-negative individuals could have negative TST/IGRA test (42). A meta-analysis of published literature revealed that IGRAs have a sensitivity of ~70-90% for the diagnosis of *active* TB disease, and it may be still lower in high endemic settings, and with advanced age (43). Furthermore, in CNS TB, up to two thirds of possible TB cases could be IGRA negative (29). Hence, it is possible that a significant number of ocular TB patients will also test negative for

**Figure 1:**
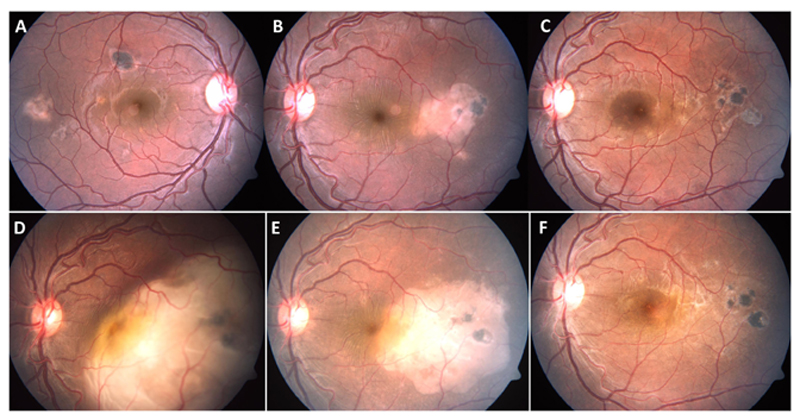
A 28-year-old immunocompetent male presented with yellowish white subretinal lesions temporal to macula in both eyes **(A, B)**. The patient had negative tuberculin skin test, chest X-ray and tests for syphilis. He was treated with a course of oral corticosteroids, tapered over four months. The lesions resolved completely in both eyes **(C)**, left eye). The patient presented eight months after completion of treatment with rapid loss of vision in left eye. The left eye fundus showed large choroidal granuloma, eight disc diameters in size, with overlying serous detachment **(D)**. The right eye did not have any active lesions. The patient, a migrant labour, reported tuberculosis (TB) contact two years ago. He was not further investigated (though warranted) and started on four drug anti-TB therapy (without any corticosteroids). There was marked decrease in size of the lesion within just two weeks of therapy **(E)**, and near total resolution of the lesion, after five weeks **(F)**.

TB immunoreactivity. Figure 1 describes one such case that was misdiagnosed on the basis of a negative TST result at initial presentation. In such situations, one option could be testing both TST and IGRA in every ocular TB suspect, as recommended by the COTS guidelines (44, 45). Those who test negative for both TST and IGRA despite strong clinical suspicion, should be followed up closely for persistent or recurrent inflammation, and retested at appropriate timepoints.

## Way Forward

Ocular TB is likely to remain a clinical diagnosis, at least in the near future, despite ongoing research on the molecular diagnosis of this condition. This is not unusual for diseases that lack a gold standard (46). For example, case definitions based on composite clinical criteria are routinely used in other paucibacillary forms of TB such as childhood and central nervous system TB (47, 48). The primary goal of these criteria is to ensure early recognition of the disease and timely initiation of ATT. The recent COTS consensus guidelines for the initiation of ATT in various forms of ocular TB, are a step in that direction (44, 45). Additional studies are required to validate the role of ATT prospectively and to determine the appropriate duration of ATT. There is also a need to differentiate between different clinical phenotypes of ocular TB, based on morphological appearance (e.g., choroidal granuloma, serpiginous-like choroiditis and retinal vasculitis) or between acute, chronic and recurrent disease. It is possible that each of these could have different pathomechanisms and therefore, different diagnostic and therapeutic requirements.

In conclusion, ocular TB and latent TB infection are mutually exclusive entities. The former is an ocular inflammatory disease requiring anti-microbial therapy, and the latter, cannot have any clinical symptoms – in the lungs or elsewhere in the body. Yet the marker of latent TB infection – TB immunoreactivity, for reasons described above, is critical to the diagnosis of ocular TB. The tests for TB immunoreactivity – TST and IGRA, should however be interpreted in the context of the clinical scenario.

## Figures and Tables

**Table 1:** Myths and facts surrounding TB immunoreactivity.

	Myth	Fact
**bacille Calmette-Gue:rin (BCG) vaccination**	BCG vaccination is a common cause of false positive tuberculin skin test (TST).	BCG vaccination in infancy has minimal effect on tuberculin skin test (TST) ≥10 years age (only 1% have TST≥10mm) (35).
**Size of TST response**	The likelihood of pulmonary TB is directly related to the size of TST response.	In the context of pulmonary tuberculosis (TB), beyond 5 mm induration, the size of induration is comparable between active TB, inactive TB, close contacts, and normal individuals. It is also unrelated to type and extent of radiographic findings (36).
**Repeat TST**	Retesting leads to increase in tuberculin reaction even in the absence of new infection.	The booster effect of repeat TST is maximal if the interval between the two tests is 1-5 weeks, and minimal if less than 48 hours or more than 60 days (37). This booster effect should be distinguished from random variability between tests (reading, biological variation), and from spontaneous conversion and reversion.
**TST vs interferon-gamma release assays (IGRA) in the diagnosis of ocular TB**	IGRA performs better than TST in the diagnosis of ocular TB.	Neither TST nor IGRA could predict the development of active TB, in high-endemic settings (38). However, discordance between IGRA and TST is common and could be influenced by ethnicity, age, and type of uveitis (39).
**Effect of anti-TB therapy on TB immunoreactivity**	Anti-TB therapy outcomes can be measured by serial testing of TB immunoreactivity.	Serial IGRA testing has no value in monitoring effect of anti-TB therapy, primarily due to within-subject variability during the course of treatment (40, 41).

## Box 1 Glossary of terms *(non-alphabetical).*

**TB immunoreactivity**: Indirect evidence of present or past infection with *Mycobacterium tuberculosis (Mtb)* as inferred by a detectable adaptive immune response to *Mtb* antigens (on tuberculin skin test or interferon gamma release assay) (6).

**Latent TB**: State of persistent immune response to stimulation by mycobacterial antigens without evidence of clinically active manifest TB – in the lungs, or any other extrapulmonary organ (including the eye) (10).

**Active TB**: Evidence of progressive disease of the lung and/or other organs generally accompanied by a positive culture for *Mtb* and/or roentgenographic findings and/or histopathology consistent with TB (11).

**Systemic infection**: Ongoing *Mtb* infection in lungs and/or extrapulmonary organs (excluding the eye for the purpose of this discussion) that may or may not be associated with clinically active manifest disease.

## Box 2 Proportion of different types of evidence of *Mycobacterium tuberculosis* infection (in color), available in in patients with and without ocular TB disease.

**Explanation**

1. **Microbiological evidence in the eye**: There is no real epidemiological data available on the proportion of patients with microbiological evidence of acid-fast bacilli in ocular *tissues*, since this investigation is rarely done in patients with ocular TB.
2. **Active systemic TB** *(including both pulmonary and extra-pulmonary TB):* This data is drawn from the Collaborative Ocular TB Study (COTS) in which symptoms suggestive of TB (chronic cough, hemoptysis, weight loss and night sweats) were found in only 8% of patients (28). An additional 23.3% had a past history of pulmonary or extrapulmonary TB.
3. **Healed systemic TB**: Again, drawing from the COTS data, healed or inactive pulmonary TB was found on chest computed tomography scans in 68.6% patients (28). The number of ocular TB patients with healed/inactive *extra-pulmonary* TB remains unknown.
4. **TB immunoreactivity**: While a positive TB immunoreactivity test is currently considered *sine qua non* for the diagnosis of ocular TB, a significant number of true ocular TB patients (data unavailable) may test negative (Figure 1). 28.8% of patients with extrapulmonary TB tested negative for interferon gamma release assay (IGRA) (29), and 40% of histopathologically proven ocular TB patients had negative tuberculin test (19). Conversely, many patients with non-TB uveitis can have positive TB immunoreactivity, the number depending on TB endemicity in the population. When tested in *all uveitis patients*, the IGRA positivity ranged from 14.4% in the United States (13), to 36% in Thailand (30), in both cases being higher than the IGRA positivity in the respective general populations. Thus, a significant number of patients without ocular TB, can test positive for TB-immunoreactivity.
